# Correction: H3K27ac-induced lncRNA PAXIP1-AS1 promotes cell proliferation, migration, EMT and apoptosis in ovarian cancer by targeting miR-6744-5p/PCBP2 axis

**DOI:** 10.1186/s13048-022-01038-5

**Published:** 2022-09-20

**Authors:** Yimin Ma, Wei Zheng

**Affiliations:** 1 Department of Gynecology, Ningbo Medical Center Lihuili Hospital, Ningbo, 315040 Zhejiang China; 2 Department of Gynecology, Xi’an Military Industry Hospital, Xi’an, 710065 Shaanxi China

**Correction: J Ovarian Res 14, 76 (2021)**

10.1186/s13048-021-00822-z

Following publication of the original article [1], the authors identified an error in Figs. 1 and 5. The correct figures are shown in the following pages.

**Fig. 1:**
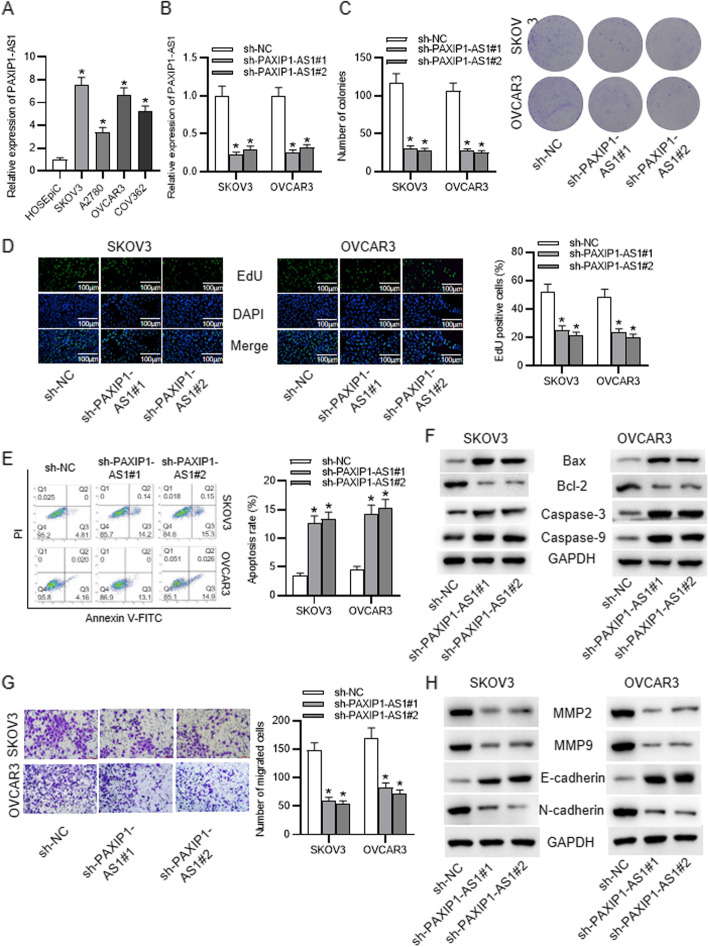
Expression pattern and functional role of PAXIP1-AS1 in OC cells. **a** RT-qPCR data of PAXIP1-AS1 expression in HOSEpiC cell line and OC cell lines. **b** Knockdown of PAXIP1-AS1 in SKOV3 and OVCAR3 cells validated by RT-qPCR. **c**-**d** Proliferation of SKOV3 and OVCAR3 cells upon PAXIP1-AS1 silencing was evaluated via colony formation assay and EdU assay. **e** Apoptosis of SKOV3 and OVCAR3 cells after PAXIP1-AS1 silencing was assessed through flow cytometry analysis. **f** Protein levels of Bax, Bcl-2, caspase-3 and caspase-9 under sh-PAXIP1-AS1 transfection were detected by western blot. **g** Migration of SKOV3 and OVCAR3 cells transfected with sh-PAXIP1-AS1 was confirmed by Transwell assay. **h** MMP2, MMP9, E-cadherin and N-cadherin protein levels were testified with western blot upon PAXIP1-AS1 knockdown. *p < 0.05

**Fig. 5:**
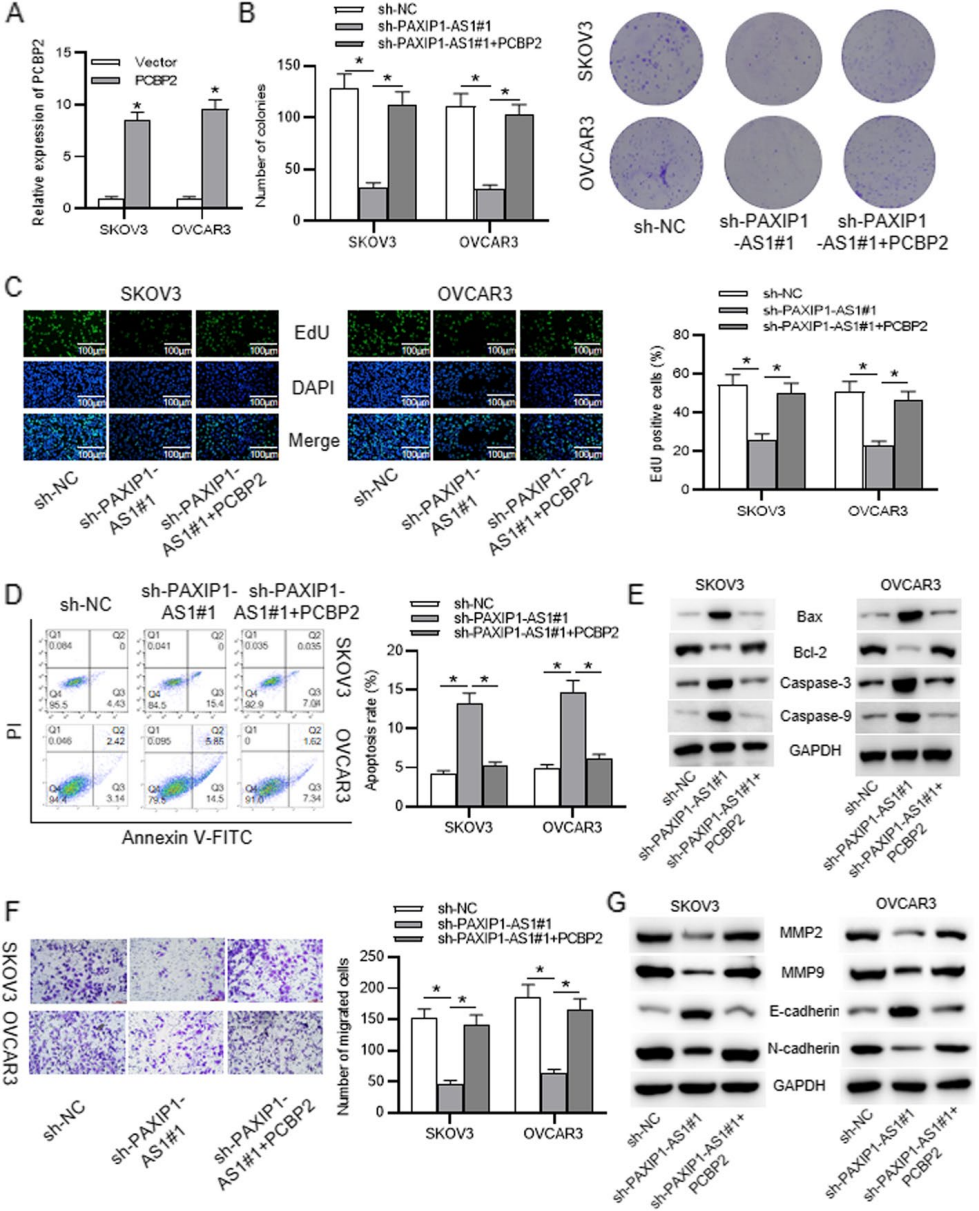
PCBP2 was a target of PAXIP1-AS1 in regulating OC cellular process. **a** Expression of PCBP2 in cells transfected with pcDNA3.1/PCBP2. **b**-**c** Cell proliferation with indicated transfection was tested by colony formation and EdU assays. **d**-**e** Apoptotic rate and levels of apoptosis-relevant proteins were respectively determined by flow cytometry analysis and western blot. **f** Cell migration in each group was measured through Transwell assay. **g** Levels of migration-related proteins and EMT-associated proteins in cells transfected with appointed plasmids were evaluated using western blot. *p < 0.05
